# Advanced computational modeling for *in vitro* nanomaterial dosimetry

**DOI:** 10.1186/s12989-015-0109-1

**Published:** 2015-10-24

**Authors:** Glen M. DeLoid, Joel M. Cohen, Georgios Pyrgiotakis, Sandra V. Pirela, Anoop Pal, Jiying Liu, Jelena Srebric, Philip Demokritou

**Affiliations:** Center for Nanotechnology and Nanotoxicology, Department of Environmental Health, Harvard T.H. Chan School of Public Health, 655 Huntington Ave, Boston, MA 02115 USA; Department of Architectural Engineering, The Pennsylvania State University, University Park, PA 16802 USA; School of Thermal Engineering, Shandong Jianzhu University, 1000 Fengming Rd, Jinan, China; Department of Mechanical Engineering, University of Maryland, College Park, MD 20742 USA

**Keywords:** Engineered nanomaterial, Nanotoxicology, *in vitro* dosimetry, Nanosafety, Hazard ranking, Fate and transport modeling

## Abstract

**Background:**

Accurate and meaningful dose metrics are a basic requirement for *in vitro* screening to assess potential health risks of engineered nanomaterials (ENMs). Correctly and consistently quantifying what cells “see,” during an *in vitro* exposure requires standardized preparation of stable ENM suspensions, accurate characterizatoin of agglomerate sizes and effective densities, and predictive modeling of mass transport. Earlier transport models provided a marked improvement over administered concentration or total mass, but included assumptions that could produce sizable inaccuracies, most notably that all particles at the bottom of the well are adsorbed or taken up by cells, which would drive transport downward, resulting in overestimation of deposition.

**Methods:**

Here we present development, validation and results of two robust computational transport models. Both three-dimensional computational fluid dynamics (CFD) and a newly-developed one-dimensional Distorted Grid (DG) model were used to estimate delivered dose metrics for industry-relevant metal oxide ENMs suspended in culture media. Both models allow simultaneous modeling of full size distributions for polydisperse ENM suspensions, and provide deposition metrics as well as concentration metrics over the extent of the well. The DG model also emulates the biokinetics at the particle-cell interface using a Langmuir isotherm, governed by a user-defined dissociation constant, *K*_D_, and allows modeling of ENM dissolution over time.

**Results:**

Dose metrics predicted by the two models were in remarkably close agreement. The DG model was also validated by quantitative analysis of flash-frozen, cryosectioned columns of ENM suspensions. Results of simulations based on agglomerate size distributions differed substantially from those obtained using mean sizes. The effect of cellular adsorption on delivered dose was negligible for *K*_D_ values consistent with non-specific binding (> 1 nM), whereas smaller values (≤ 1 nM) typical of specific high-affinity binding resulted in faster and eventual complete deposition of material.

**Conclusions:**

The advanced models presented provide practical and robust tools for obtaining accurate dose metrics and concentration profiles across the well, for high-throughput screening of ENMs. The DG model allows rapid modeling that accommodates polydispersity, dissolution, and adsorption. Result of adsorption studies suggest that a reflective lower boundary condition is appropriate for modeling most *in vitro* ENM exposures.

**Electronic supplementary material:**

The online version of this article (doi:10.1186/s12989-015-0109-1) contains supplementary material, which is available to authorized users.

## Background

Assessing the potential health risks associated with exposures to the vast number and variety of engineered nanomaterials (ENMs) entering manufacturing workplaces and now present in myriad consumer products is a daunting task that requires fast, inexpensive and reliable screening strategies [1–5]. Although animal testing may provide the most relevant and reliable assessment of a nanomaterial’s biological activity, the associated cost and throughput constraints, as well as humane concerns, render this approach impractical [1, 4, 6]. A screening approach using *in vitro* cell-based assays provides a logical and efficient alternative [7]. However, *in vitro* testing has often produced results that are inconsistent with those of corresponding *in vivo* studies and even of other *in vitro* studies of the same ENMs [4–8]. Although the many differences between monocultured cell and whole animal experimental systems may account for much of this disparity, it is likely that failure to match *in vitro* and *in vivo* doses, as a result of inadequate characterization of ENM powders and suspensions, and more importantly, failure to account for transport of ENM particles or agglomerates in suspension over time, is in part responsible for these disparities [9–13].

Until recently most *in vitro* studies have reported dose in terms of either an initial administered mass concentration or of a total administered mass [11, 14, 15]. The former assumes that sedimentation either does not occur, or is negligible, and the latter assumes that it is complete, with all of the suspended material instantly transported to the cells at the bottom of the cell culture well. The reality lies between these extremes, and depends upon the intrinsic physicochemical properties of the suspended material, the extrinsic properties of suspending media, and the time course of the exposure.

**Fig. 1:**
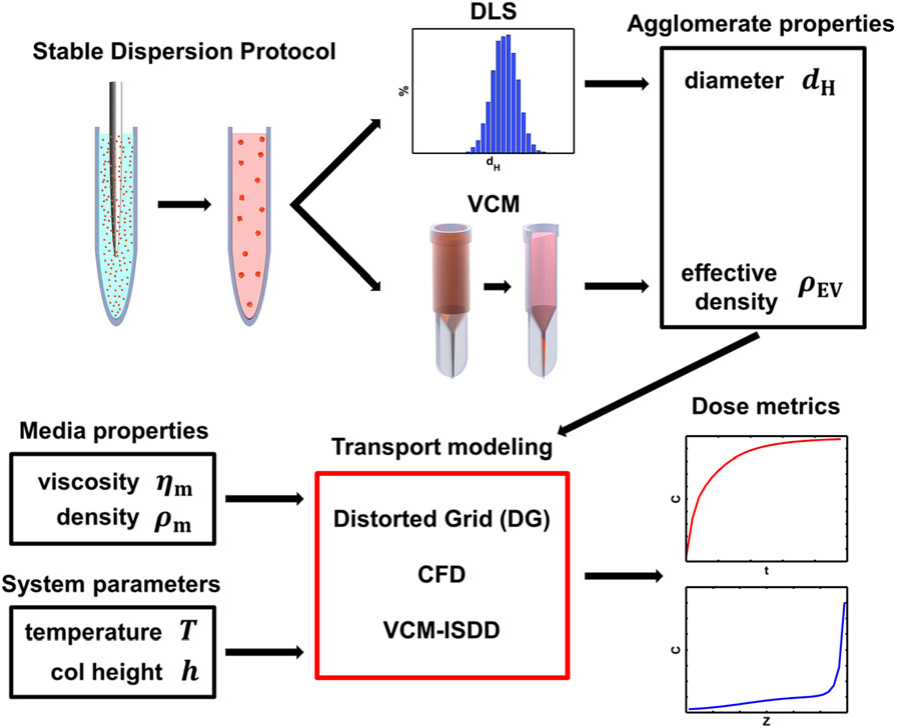
Integrated nanodosimetry approach overview. Methodologies required for accurate nano-dosimetry include: 1) standardized dispersion preparation protocols, 2) detailed colloidal suspension characterization including size and effective density of formed agglomerates, and 3) computational modeling of transport based on agglomerate, media and system properties. Standardized dispersion protocol to maximize stability of agglomeration state includes sonication of nanomaterial in deionized water to particle-specific critical dispersion sonication energy (*DSE*_cr_), followed by dilution into final application media. Dispersions are analyzed by DLS to determine agglomerate hydrodynamic diameters, and by VCM to determine agglomerate effective density. Transport modeling to determine dose metrics requires *d*_H_ from DLS and *ρ*_EV_ from VCM, as well as media properties (viscosity, *η*_m_ and density, *ρ*_m_) and system parameters (temperature, Τ and media column height, *h*). Available computational transport models include VCM-ISDD, computational fluid dynamics (CFD) and Distorted Grid (DG, introduced in this report) models. Possible output dose metrics include exposure concentrations in the cell microenvironment at the bottom of the well (including mass, surface area and particle number), fractional or absolute deposition (in terms of mass, surface area and particle number), as well as concentration as a function of vertical position within the well (concentration profile)

Of late, greater emphasis has been placed upon achieving a better understanding of the exposures experienced by cells *in vitro*, and of the measures necessary for accurate and meaningful dosimetry [12, 16–20] (+1 for > =20). These include several standardized methodologies, from generation of stable suspensions, to accurate physical characterization of formed agglomerates in suspension, to appropriate modeling of the transport of particles or agglomerates during exposure. An integrated approach for *in vitro* nanodosimetry based on these methodologies is depicted in Fig. 1.

Most industrially-relevant ENMs form agglomerates when dispersed in liquid. The size, or size distribution, and effective density of these agglomerates determine their transport properties (sedimentation and diffusion coefficients), and thus control the rate at which the material settles, which in turn determines the ENM mass, surface and particle number delivered to cells as a function of time [12, 16, 17]. ENM agglomeration state can also influence the nature and extent of biological effects [21, 22]. As we have recently reported, the agglomerate size distribution, and more importantly the agglomerate stability (re-agglomeration rate), are dependent upon the dispersion protocol and properties of the dispersant [11, 17]. Initial sonication of ENM powder in deionized water at or above an ENM-dependent critical delivered sonication energy (*DSE*_cr_), followed by dilution into culture media, produces suspensions with remarkably stable size distributions, whereas the size distributions of suspensions created at less than *DSE*_cr_ can change substantially (suggesting re-agglomeration) over time [11].

Once a stable suspension is created, particle sizes are typically determined by either dynamic light scattering (DLS), disk centrifugation, or tunable resistive pulse sensing technology (TRPS) [13] and are routinely reported in *in vitro* studies. Effective density of agglomerates can differ from bulk ENM density by many fold due to the formation of protein coronae and intra-agglomerate trapping of media [12, 17]. Despite its important influence on transport and thereby on delivered cellular dose, it is rarely measured and reported in published *in vitro* studies. This is primarily due to methodological limitations that until recently required expensive instrumentation such as an Analytical Ultracentrifuge (AUC). The volumetric centrifugation method (VCM), recently developed by the authors, provides a fast and practical alternative for determination of effective density of ENMs in suspension [12].

The first computational fate and transport model developed for ENM dosimetry was the In vitro Sedimentation, Diffusion and Dosimetry model (ISDD) reported by Hinderliter et al. [16]. The ISDD model utilizes an analytical solution of the Mason Weaver equation to determine, in a suspension column of a given height, and a given concentration of particles or agglomerates of a given diameter and density, the per area mass, surface area, and number of particles, as well as the fraction of total suspended material deposited as a function of time. This provided a ground-breaking improvement in dosimetry accuracy and enabled meaningful comparisons and safety hazard rankings among ENMs of different physiochemical properties. The ISDD initially estimated effective density using the Sterling equation, based on a theoretical fractal model and estimated fractal dimensions for ENM agglomerates [23]. More recently the ISDD has been modified to utilize the empirically-based and more accurate VCM to measure effective density, with the integrated approach now referred to as VCM-ISDD [17].

Although the VCM-ISDD model importantly allows assessment of relative deposition over time, it employs a perfectly adsorptive (sticky) lower boundary condition, the result of which is the prediction of complete deposition for all materials, within a few hours to several days, depending on particle size and density. This is inconsistent with our observations of nanoparticle suspensions over extended periods of time (months). With the exception of cases in which particles are sufficiently large or dense that the role of diffusion relative to sedimentation is negligible, or cases in which strong adsorption or uptake by cells creates a perpetual sink at the bottom of the well, the concentration gradient created by sedimentation would drive net diffusion upward, such that an equilibrium is eventually obtained, and complete deposition would not occur. The VCM-ISDD model is also limited to modeling transport of a single agglomerate size at a time. Although transport of a polydisperse suspension can be modeled by summing results of individual simulations for each size category, this is unwieldy for high-resolution size distributions, which from DLS, for example, may contain up to 300 size species, and moreover it precludes the possibility of adjusting for dynamic cross-species interactions and effects on transport parameters. In addition, output from the VCM-ISDD is limited to the absolute or fractional deposition over time, and does not provide concentration metrics, which more closely represent what cells “see.” Finally, the VCM-ISDD does not provide mechanisms for modeling of ENMs that undergo dissolution with time, which can not only reduce deposition of solid ENM and add a dissolved dose component, but may also dynamically change relevant transport parameters of particles. Thus although the VCM-ISDD provides useful measures and relative comparisons of transport and effective dose among different materials, a number of refinements are needed.

Here we present results from two advanced numerical transport models that address these issues: 1) a new one-dimensional iterative distorted grid (DG) model, and 2) the gold standard three-dimensional computational fluid dynamics (CFD) approach. Each of these models provide deposition as well as concentration of ENMs as a function of time, both at the bottom of a culture well (the cell microenvironment) and as a function of vertical position within suspension column. Both models accommodate simultaneous simulation of polydisperse suspensions. The DG model further provides modeling of particles that undergo dissolution over time, as well as a variable ‘stickiness’ boundary condition at the bottom, implemented using a Langmuir isotherm process. As we demonstrate below, the results of the distorted grid model, which was implemented in MATLAB, and typically runs in a few minutes on standard desktop computer, are in close agreement with those from CFD. Finally, we validated the DG model by experimentally measuring particle deposition profiles in thinly-sectioned frozen columns of industry-relevant ENM suspensions.

## Results

### Comparison of Distorted Grid and CFD model simulations

The Distorted Grid (DG) model is based on a model previously developed for analysis of protein systems via their behavior in an ultracentrifuge [24–30], which was adapted for particle transport and implemented in MATLAB. In this model the column of suspended particles is divided into *n* compartments separated by *n* + 1 boundaries, and successive brief rounds of simulated sedimentation and diffusion are performed, with transfer of suspended material between compartments. A detailed mathematical description of the DG model design is provided in the methods section.

In the Computational Fluid Dynamics (CFD) model, particles are initially assigned to compartments of a 3-dimensional grid representation of the suspension column, and a solution of the Navier–Stokes equation is used to calculate the movement of individual particles between compartments. A complete description of the CFD model used in these studies is presented in methods.

**Fig. 2:**
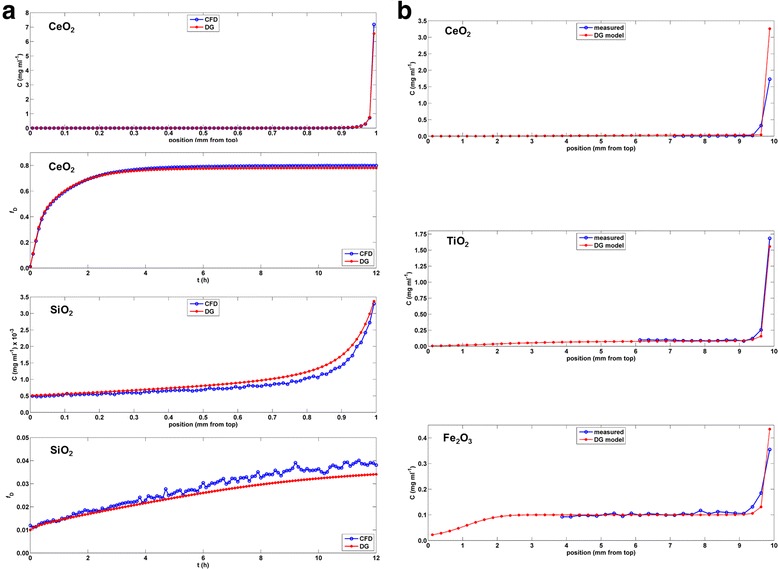
Distorted Grid model cryosection validation. **a** Concentration profiles and material fraction deposited (present in the bottom 10 μm of the cell) predicted by the DG model are compared with those obtained from CFD for CeO_2_ and SiO_2_. (*C*_0_ = 0. 1 mg ml^−1^ for CeO_2_ and 0.001 mg ml^−1^ for SiO_2_, column height = 1 mm, *d*_H_ = volume size distribution from DLS, see Additional file 1: Figure S2). **b** Concentration profiles predicted by the DG model are compared with those obtained from corresponding cryosectioned samples for CeO_2_ (24 h), TiO_2_ (64 h) and Fe_2_O_3_ (24 h) nanomaterials (*C*_0_ = 0.1 mg ml^−1^, column height = 10 mm, *d*_H_ = volume size distribution from DLS (Additional file 1: Figure S2))

Concentration profiles (ENM mass concentrations as a function of distance from the bottom of the well) at 12 h, and fractional deposition (fraction of mass present in the bottom 10 μm of the well) as a function of time, as computed by the DG and CFD models, for CeO_2_(in RPMI + 0.5 % BSA) and SiO_2_ (in PBS + 0.1 % BSA), are compared in Fig 2a. A thickness of 10 μm was somewhat arbitrarily chosen for the disk at the bottom of the well representing the cell microenvironment. We based this choice on thickness typically observed for typical adherent cells in culture. However both the DG and CFD models allow this thickness value to be specified by the user, and smaller or larger thicknesses may be appropriate depending on the type of cell or system being studied. Properties of ENM powders and of their forms in liquid suspension, including effective densities (*ρ*_EV_), are given in Table 1. The volume-weighted size (*d*_H_) distributions (calculated from particle number distributions from DLS as described in methods) of liquid suspensions used in these simulations are shown in Additional file 1: Figure S3.

**Table 1:** ENM properties

Material	SSA (m^2^ g^−1^)	*d*_BET_ (nm)	Media	*d*_Hv_ (nm)	PdI	*ρ*_ENM_ (g cm^−3^)	*ρ*_EV_ (g cm^−3^)
VENGES SiO_2_	147	18.6	PBS + 0.1 % BSA	149.9	0.175 ± 0.013	2.648	1.564 ± 0.009
VENGES Fe_2_O_3_	41.5	27.6	RPMI + 10 % HS	234.5	0.755 ± 0.203	5.242	1.335 ± 0.000
VENGES CeO_2_	144	5.4	RPMI + 0.5 % BSA	982.1	0.310 ± 0.091	7.215	1.420 ± 0.004
EVONIK TiO_2_	50	21	RPMI + 10 % HS	397.8	0.233 ± 0.018	4.230	1.251 ± 0.005
Alfa Aesar ZnO	17	63	RPMI + 10 % FBS	307.0	0.303 ± 0.122	5.606	1.650 ± 0.070

SSA: (specific surface area) by nitrogen adsorption/Brunauer-Emmett-Teller (BET) method. *d*_BET_ : primary particle diameter determined from *SSA*, *d*_XRD_ : particle diameter as determined by X-ray diffraction, <*d*_H_ > _v_: mean hydrodynamic diameter from DLS volume distribution, *ρ*_ENM_: bulk ENM material density, *ρ*_EV_: effective density estimated by volumetric centrifugation.

Simulation results for CFD and DG results for CeO_2_ and TiO_2_ are nearly identical (within <5 %) (Fig. 2). Because the CFD model tracks movement of each individual particle it is highly computationally-intensive, which places practical constraints on the number of particles, and thus the model geometry and concentration of ENMs, that can be simulated in a reasonable time with available computational resources. Thus, in our CFD simulations we employed a column height of 1 mm, and concentrations of 0.1 mg ml^−1^ for CeO_2_ and 0.001 mg ml^−1^ for SiO_2_, resulting in a total of approximately 3.8 × 10^5^ particles in each case. Even at these low values the CFD simulations for CeO_2_ and SiO_2_ required, respectively, 12 and 48 h of compute time on a multi-processor supercomputer. By contrast, the corresponding simulations using the DG model were each completed in less than a minute on a standard single-processor PC.

The results of both the DG model and CFD can be affected by selection of compartment (DG) or grid (CFD) size. To ensure grid size-independence, simulations were performed over a range of compartment sizes for SiO_2_. Whereas the final predicted deposition or bottom of column concentration increased substantially as compartment and grid sizes were decreased from 50.0 to 5.0 μm, results were independent of compartment/grid sizes for values less than 5 μm (data not shown). Likewise, simulations were run over a range of iteration time intervals to verify that results were independent of time interval between 0.1 to 1.0 seconds.

### Experimental Validation of Distorted Grid model by frozen section analysis

Concentration profiles predicted by the DG model were compared with those obtained from frozen sections of corresponding columns of nanoparticle suspensions. Columns of Fe_2_O_3_, CeO_2_ and TiO_2_ suspensions (powder and liquid suspension properties shown in Table 1) were flash frozen and cryosectioned following incubations (to allow transport) at room temperature of 24 h for Fe_2_O_3_ and CeO_2_, and 64 h for TiO_2_. Material concentrations within individual sections were determined by spectrophotometry using standard curves prepared from frozen-and-thawed suspensions of known mass concentrations. The process for creating suspension columns and for obtaining cryosectioned slice concentrations is described in the methods section and illustrated in Additional file 1: Figure S2. For all three ENMs tested the concentration profile predicted by the DG model was in close agreement with that obtained from frozen sections (Fig. 2b).

### DG model-derived mass, particle number and surface dose metrics

Although ENM mass concentration and deposition per unit area are the most commonly-used *in vitro* dose metrics, it is also important to consider exposures in terms of particle number and surface area [9, 10, 15]. Not only is it possible that biological effects may in some cases track more closely with these values, but because of differences in agglomerate formation the relative magnitudes of number and surface area metrics for suspensions of different materials undergoing transport may differ substantially from the corresponding mass metrics. The DG model provides both concentration and deposition outputs in terms of number of particles (N ml^−1^ and N cm^−2^) and ENM surface area (cm^2^ ml^−1^ and cm^2^ cm^−2^). Calculation of number and surface area metrics from mass concentration and deposition metrics are described in methods.

**Fig. 3:**
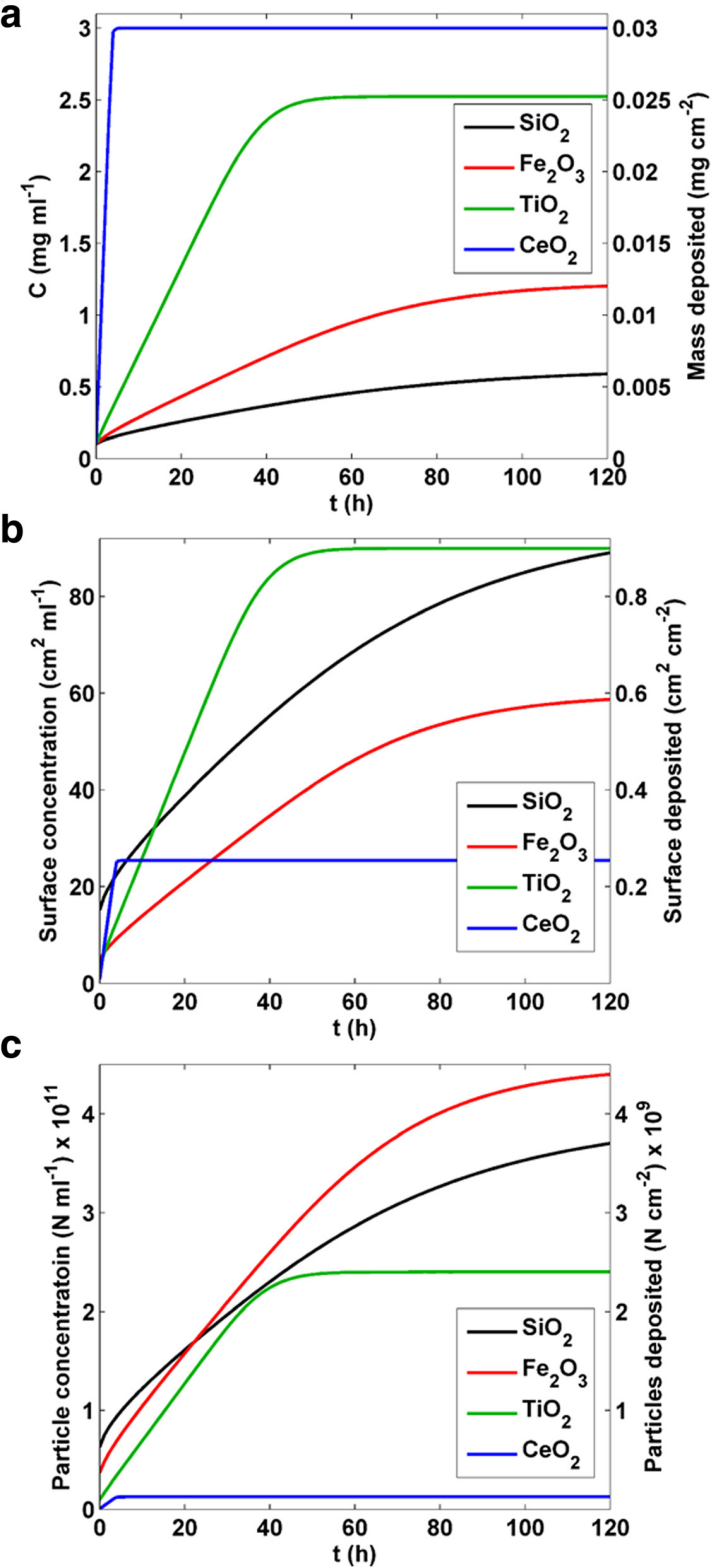
DG model output dose metrics. DG model dose metric output data (in bottom 10 μm) for SiO_2_, Fe_2_O_3_, TiO_2_ and CeO_2_ nanomaterials (*C*_0_ = 0.1 mg ml^−1^, column height = 3 mm, *d*_H_ = volume-averaged mean (Table 1), reflective boundary condition) from 0 to 120 h. **a** ENM mass concentration (mg ml^−1^) and mass deposited per well bottom surface area (mg cm^−2^). **b** ENM surface area concentration (cm^2^ ml^−1^) and ENM surface area deposited per unit floor surface area (cm^2^ cm^−2^). **c** Particle number concentration (N ml^−1^) and particle number deposited per unit area (N cm^−2^)

DG model-derived mass, particle number and ENM surface area dose metrics as a function of time (from 0 to 120 h) for four ENMs (SiO_2_, Fe_2_O_3_, TiO_2_ and CeO_2_), with initial mass concentration, *C*_0_ = 0.1 mg ml^−1^, suspension column height of 3 mm, and volume averaged particle diameter (〈*d*_H_〉_v_ x, Table 1) are shown in Fig. 3. Because we define deposition (not equivalent to binding, which is discussed below) as the total mass, particle number or surface area in the bottom 10 μm of the column, it is calculated as the product of concentration and the volume of that disk. The shapes of the concentration curves are therefore identical to those of the corresponding deposition curves, differing only in scale and units, and both can be represented in the same graph using appropriately scaled axes (Fig. 3). Interestingly, the relative magnitudes and ranking of the four materials by concentration (or deposition) differs for the three different classes of metrics. For example, mass concentration and deposition are at all time points greatest for CeO_2_ and smallest for SiO_2_, whereas particle number and surface area concentration and deposition of CeO_2_ are much less than those of SiO_2_. These differences are not a result of transport differences *per se*, but are rather due to differences in agglomeration state. Specifically, since the agglomerates of SiO_2_ (〈*d*_H_〉_v_ =149.9 nm) are much smaller than those of CeO_2_ (〈*d*_H_〉_v_ = 982.1 nm), the total number and surface area of SiO_2_ for a given mass concentration are considerably greater than the corresponding values for CeO_2_.

### Effect of particle-cell binding/adsorption – well bottom boundary condition

The data presented to this point, including those from validation experiments, were obtained using a reflective boundary condition at the bottom of the well: particles arriving at the bottom were not bound or removed from the system, but remained present, undergoing Brownian motion, and contributing to the concentration gradient driving diffusion. The opposite extreme boundary condition is one in which all particles that reach the bottom of the well adhere to the cells there, effectively removing them from the concentration gradient. The real world condition likely lies somewhere between these two extremes, and is ENM-, media-, and cell type-dependent. Some of the ENM agglomerates that reach the cell microenvironment in an *in vitro* exposure system may adhere to cells, or in the case of phagocytic cells may be internalized, which in either case would eliminate their contribution to the diffusion gradient opposing sedimentation, and thereby increase the net rate of transport toward the bottom of the well. Quantification of particle-cell binding and uptake is an emerging research area [22] and a complex topic that will be addressed in future work, but because binding can, as we will demonstrate, have a large effect on net transport, it is important to incorporate this variable in fate and transport models. Initially we can employ reasonable estimates of binding, based on our understanding of the particle- and protein-protein interactions that are likely to be involved, which can be later refined as specific data for particle-cell binding kinetics become available. Accordingly, an adsorption strength (stickiness) parameter was incorporated in the DG model to simulate boundary conditions with variable levels of adsorption at the bottom of the well. This is implemented as a Langmuir isotherm process [31], in which adsorption is characterized by the equilibrium dissociation constant, *K*_D_, as described in the methods section.

**Fig. 4:**
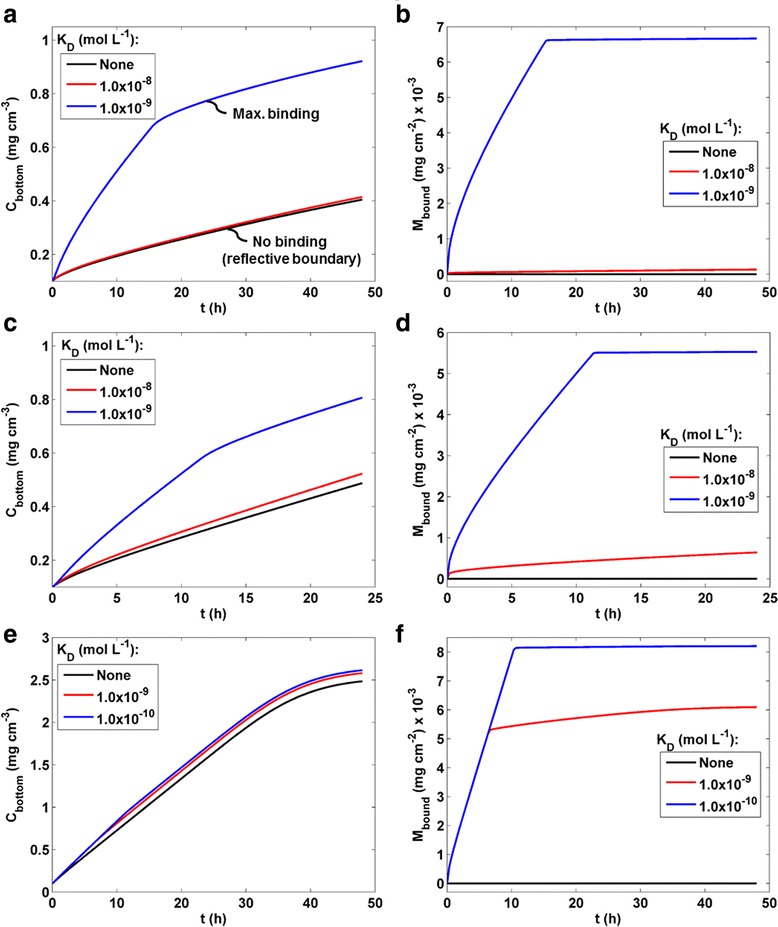
Effect of binding on DG-predicted dose metrics. Effect of particle/agglomerate binding to cells (well bottom) on DG model dose metrics for SiO2 (**a**, **b**), Fe_2_O_3_ (**c**, **d**), and TiO2 (**e**, **f**). For all simulations *C*_0_ = 0.1 mg ml^−1^, column height = 3 mm, and *d*_H_ = volume-averaged mean (Table 1). Langmuir isotherm adsorption was used to model binding of agglomerates to the well bottom at various values of the dissociation constant *K*_D_. **a**, **c**, **e** Exposure mass ENM concentration, *C*_bottom_ (mg cm^−3^ in bottom 10 μm, including ENM within both bound and free agglomerates). **b**, **d**, **f** Mass of ENM bound per well bottom area (mg cm^−2^)

The effect of cellular adsorption on total (free + cell bound) ENM mass concentration in the lower 10 μm exposure volume, as well as the cell-bound ENM mass per unit well bottom area (mg cm^−2^) for SiO_2_, Fe_2_O_3_ , and TiO_2_ (all at *C*_0_ = 0.1 mg ml^−1^, for a 3 mm column height, using volume averaged particle diameter (〈*d*_H_〉_v_, Table 1) ), with various values of *K*_D_, are shown in Fig. 4. It is clear from these results that very small values of *K*_D_, in the nanomolar range, produce sizeable changes in exposure concentrations (at the bottom of the well). Further decreases in *K*_D_ did not increase predicted well bottom concentrations beyond those for the smallest *K*_D_ values shown. Such small values correspond to high-affinity binding typical of specific protein interactions, and are several orders of magnitude smaller than the millimolar or micromolar values typical of non-specific interactions [32–34]. Interactions between ENM agglomerates and cell surface biomolecules are most likely of the latter, weak non-specific type. We therefore assume that in most cases the effect of particle-cell binding on transport is negligible, and the reflective boundary condition was therefore used in all other simulations reported here.

Because the VCM-ISDD model imposes a perfectly sticky (sink) lower boundary condition, we would expect it to predict faster and more complete deposition of material than the DG model with a reflective boundary, particularly for smaller particles, where diffusion plays a relatively larger role. To test this we compared dose metrics predicted by the DG and VCM-ISDD models for four materials (SiO_2_, Fe_2_O_3_, TiO_2_ and CeO_2_), at initial mass concentration, *C*_0_ = 0.1 mg ml^−1^, suspension column height = 3 mm, and volume averaged particle diameter (〈*d*_H_〉_v_, Table 1). The results of these simulations are shown in Additional file 1: Figure S4. Whereas both models are in almost perfect agreement regarding the transport of CeO_2_ (〈*d*_H_〉_v_ =982.1 nm, *ρ*_EV_ =1.420 g cm^−3^), they provide slightly different predictions for TiO_2_(〈*d*_H_〉_v_ =397.8 nm, *ρ*_EV_ =1.251 g cm^−3^), and strongly different predictions for Fe_2_O_3_ (〈*d*_H_〉_v_ =234.5 nm, *ρ*_EV_ =1.335 g cm^−3^) and SiO_2_ (〈*d*_H_〉_v_ =149.9 nm, *ρ*_EV_ =1.564 g cm^−3^). Specifically, the VCM-ISDD model predicts deposition fraction approaching 1.0 for all materials, whereas the DG model predicts complete deposition only in the case of CeO_2_, which forms the largest agglomerates. For the three other materials the DG model predicts that the deposited fraction approaches an equilibrium value well below 1.0, and less than 0.2 for SiO_2_, which forms the smallest agglomerates.

### Effect of agglomerate size and density on dosimetry

The sedimentation velocity, *v*_s_, of a particle of hydrodynamic diameter *d*_H_ (m) under gravity is defined as

1 $$ {v}_{\mathrm{s}}=\frac{g\left({\rho}_{\mathrm{EV}}-{\rho}_{\mathrm{media}}\right){d}_{\mathrm{H}}^2}{18\eta } $$

where *g* is the acceleration due to gravity, *g* (m s^−2^), *ρ*_EV_ is the effective density of the particle (kg m^−3^), *ρ*_media_ is the media density (kg m^−3^), and *η* is the media dynamic viscosity (kg m^−1^ s^−1^). The rate of mass transport by diffusion is proportional to the diffusion coefficient *D* (m^2^ s^−1^), which is defined by the Stokes-Einstein equation as:

2 $$ D=\frac{k_{\mathrm{B}}T}{3\pi \eta {d}_{\mathrm{H}}} $$

in which *k*_B_ is the Boltzmann constant (kg m^2^ s^−2^ K^−1^), *T* is the absolute temperature (°K), and *η* is the media dynamic viscosity (kg m^−1^ s^−1^). Sedimentation velocity is thus proportional to the difference between the densities of the sedimenting particle and the suspending media, and to the square of particle diameter (Eq. 1), whereas diffusion is independent of density and linearly related to particle diameter (Eq. 2). Transport modeling should therefore predict that more dense and particularly larger particles concentrate more rapidly at the bottom of a column of suspension than less dense and smaller particles.

**Fig. 5:**
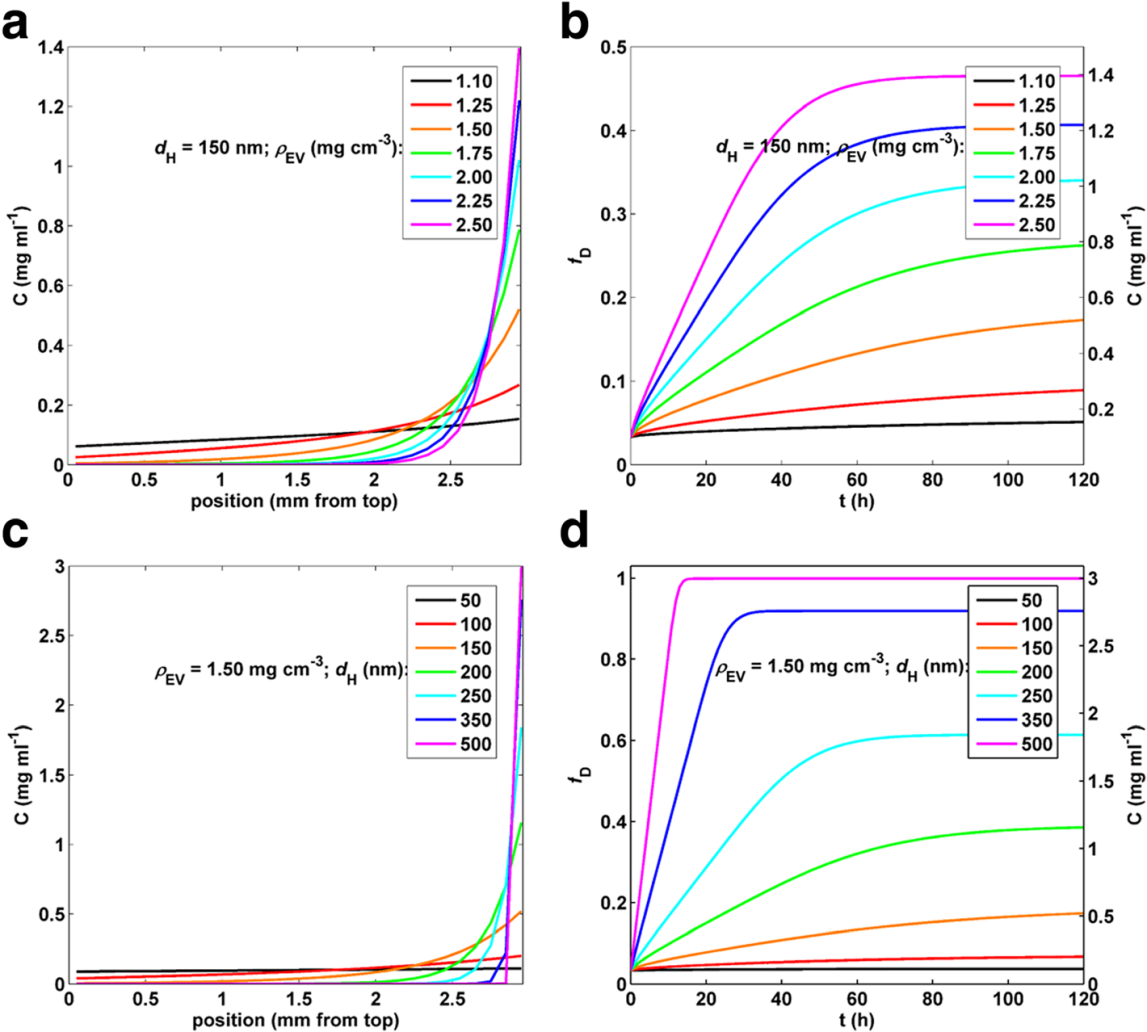
Effect of diameter and effective density on DG-predicted dose metrics. **a** and **b**, DG model output as a function of effective particle density (*ρ*_EV_ from 1.1 to 2.5 g cm^−3^) at constant *d*_H_ (150 nm). **a**, Well concentration profiles at 120 h. **b**, Fraction of material deposited and well bottom (bottom 10 μm) concentration (mg ml^−1^) from 0 to 120 h. **c**, from 0 to 120 h. **c** and **d**, DG model output as a function of particle/agglomerate diameter (*d*_H_ from 50 to 500 nm) at constant *ρ*_EV_ (1.5 g cm^−3^). **c**, Well concentration profiles at 120 h. **d**, Fraction of material deposited and well bottom (bottom 10 μm) concentration (mg ml^−1^) from 0 to 120 h

We simulated transport using the DG model for hypothetical particles of constant size (*d*_H_ = 150 nm) ranging in density from *ρ*_EV_ =1.1 to 2.5 g cm^−3^, and for particles of constant density (*ρ*_EV_ =1.5 g cm^−3^) ranging in size from *d*_H_ = 150 to 500 nm. Results of these simulations are shown in Fig. 5. As expected, for particles of constant size, predicted transport was more rapid for particles of higher density, and likewise for particles of constant density, predicted transport proceeded more rapidly for larger particles.

### Effect of Polydispersity

**Fig. 6:**
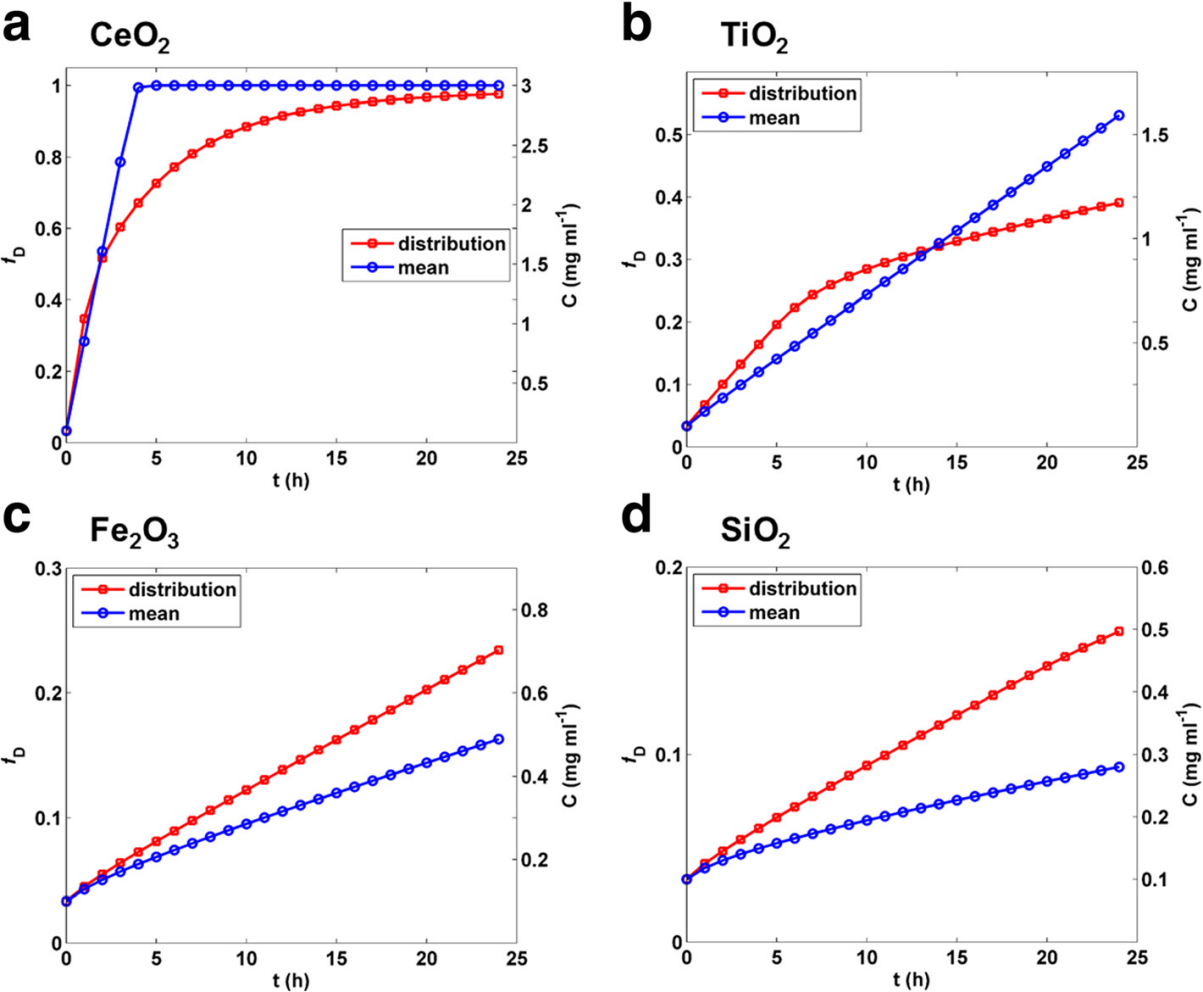
Effect of polydispersity on DG-model output. Comparison of DG-predicted fraction of mass deposited (*f*_D_) or exposure concentrations (mg ml^−1^ in bottom 10 μm of well) from 0 to 24 h for mean *d*_H_ (Table 1) and volume size distribution from DLS (Additional file 1: Figure S3) for **a**, CeO_2_, **b**, TiO_2_, **c**, Fe_2_O_3_ and **d**, SiO_2_ ENMs (*C*_0_ = 0.1 mg ml^−1^, column height = 3 mm, reflective boundary condition (no binding))

Since most ENM suspensions are polydisperse, it is important to account for the effect of polydispersity when modeling transport. The DG model allows input of a particle size distribution consisting of paired arrays of particle hydrodynamic diameters and fractional contributions to the total agglomerate mass (e.g., from DLS). We compared concentration profiles and dose metrics obtained from the DG model using volume-weighted polydisperse size/fraction arrays with those obtained using volume-weighted average sizes (*d*_Hv_), for SiO_2_, Fe_2_O_3_, TiO_2_ and CeO_2_ suspensions (24 h simulations, *C*_0_ = 0.1 mg ml^−1^, column height = 3 mm, volume averaged particle diameters shown in Table 1, volume-weighted distributions shown in Additional file 1: Figure S3). The results, shown in Fig. 6, reveal substantial differences between modeling with mean size and modeling using size/fraction arrays. In the case of CeO_2_, for example, well bottom concentration and fraction deposited reached maximum plateaus at 4 h when transport was modeled using an average diameter, but had only reached approximately two thirds maximum at the same time when modeled using the full size distribution. In the Case of TiO_2_, average size simulations resulted in higher predicted concentration and deposition at early time points, but lower predicted values at later times, relative to modeling with polydispersity. For both Fe_2_O_3_ and SiO_2,_ average size simulations substantially under-predicted concentration and deposition at all times relative to the polydisperse simulations.

### Effect of solubilization

For some nanomaterials it is important to account for solubilization of the material over time during *in vitro* exposure. Xia et al. (2008) [35] found that for 10 μg ml^−1^ (122.8 μM) suspensions of ZnO in DMEM with 10 % FBS, up to 74 % of the ZnO was solubilized within 10 minutes (corresponding to a dissolved concentration of 90 μM), and that in water initial dissolution was less extensive (20 μM), but continued linearly over 3 h to a maximum of 60 μM. Solubilization of ENM during an *in vitro* exposure not only reduces the concentration of solid ENM to which cells are exposed, and adds an exposure of solubilized or ionized material, but may also change the transport properties of agglomerate species. Although more complex relationships between solubilization and agglomerate state and agglomerate transport properties are possible, as a first approximation we modeled solubilization as a reduction of agglomerate size in proportion to the extent of dissolution.

**Fig. 7:**
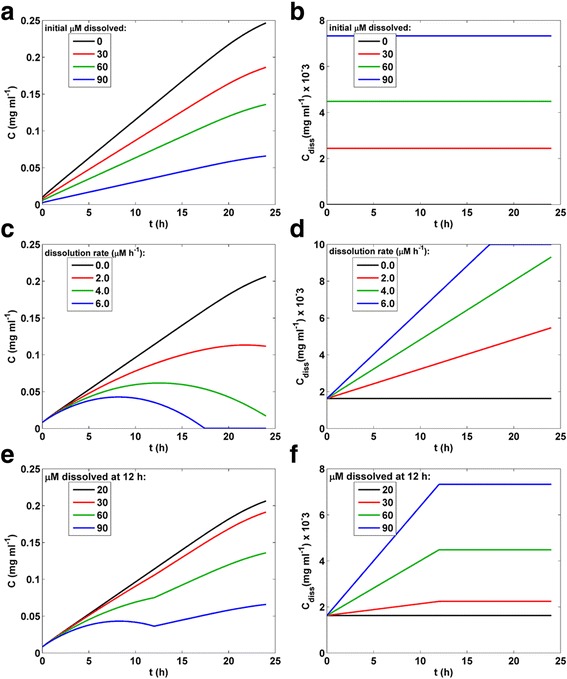
Effect of ENM solubilization on DG-predicted dose metrics. Solid ENM mass exposure concentration (in bottom 10 μm) and dissolved ENM concentrations predicted by DG model under different solubilization scenarios for ZnO (*C*_0_ = 0.01 mg ml^−1^, column height = 3 mm, *d*_H_ = volume-averaged mean (Table 1)) for 24 h. **a**, **b**, ZnO solid mass exposure concentration (mg ml^−1^ in bottom 10 μm of well) and solubilized ZnO concentration (mg ml^−1^) for initial solubilization to 0, 30, 60 and 90 μM (0.0, 2.442, 7.327 and 4.885 μg ml^−1^, dissolved fraction = 0.0, 0.2442, 0.4485, and 0.7327) with no further dissolution during transport. **c**, **d**, solid mass and dissolved exposure concentrations with initial solubility of 20 μM (1.63 μg/ml, dissolved fraction = 0.163) and continuous constant dissolution of 0, 2, 4 and 6 μM h^−1^ (0.0, 0.16, 0.32 and 0.48 μg ml^−1^ h^−1^, fraction dissolved = 0.0, 0.016, 0.032 and 0.048 h^−1^), **e**, **f**, solid and dissolved exposure mass concentrations with initial solubility of 20 μM and further linear increase in dissolution up to 12 h to maximum total dissolution of 20, 30, 60 and 90 μM (1.63, 2.442, 7.327 and 4.885 μg ml^−1^, dissolved fraction = 0.163, 0.2442, 0.4485, 0.7327) at 12 h and remaining constant after 12 h.

Solid ENM mass exposure concentration (in bottom 10 μm) and dissolved ENM concentrations predicted by DG under various solubilization scenarios for ZnO (*C*_0_ = 0.01 mg ml^−1^, column height = 3 mm, *d*_H_ = volume-averaged mean (Table 1)) for 24 h transport duration are shown in Fig. 7. ZnO solid mass exposure concentration (mg ml^−1^ in bottom 10 μm of well) and solubilized ZnO concentration (mg ml^−1^) for initial solubilization to 0, 30, 60 and 90 μM (0.0, 2.442, 7.327 and 4.885 μg ml^−1^, dissolved fraction = 0.0, 0.2442, 0.4485, and 0.7327) with no further dissolution during transport are shown in Fig. 7a and b. Solid mass and dissolved exposure concentrations with initial solubility of 20 μM (1.63 μg/ml, dissolved fraction = 0.163) and continuous constant dissolution of 0, 2, 4 and 6 μM h^−1^ (0.0, 0.16, 0.32 and 0.48 μg ml^−1^ h^−1^, fraction dissolved = 0.0, 0.016, 0.032 and 0.048 h^−1^) are shown in Fig. 7c and d. Solid and dissolved exposure mass concentrations with initial solubility of 20 μM and further linear increase in dissolution up to 12 h to maximum total dissolution of 20, 30, 60 and 90 μM (1.63, 2.442, 7.327 and 4.885 μg ml^−1^, dissolved fraction = 0.163, 0.2442, 0.4485, 0.7327) at 12 h, and remaining constant thereafter, are shown in Fig. 7e and f.

## Discussion

We have experimentally validated the predictions of the DG model by analysis of frozen and sectioned columns of test materials, and shown that the results of the DG model compare closely with the CFD model. Although CFD may be considered the gold standard for transport modeling, it is computationally-intensive, requiring expensive software and hardware, and long compute times, whereas the DG model requires only MATLAB, and a typical personal computer, and runs in a few minutes or less.

We have demonstrated here that the suspension-cell boundary condition at the bottom of the well is a critical determinant of predicted transport and dose. With a reflective boundary, sedimentation generates a gradient that drives diffusion upward, which reducing net downward transport and leading to an equilibrium gradient beyond which no further net transport occurs. Conversely, when the boundary is modeled as perfectly adherent, particles are sequestered to a greater or lesser extent depending on the value of *K*_D_, reducing, and in the extreme case inverting the diffusion-driving gradient, and thus increases net transport toward the bottom. Our results show that values of *K*_D_ in the nanomolar range or lower are required to appreciably affect delivered dose metrics (Fig. 4). *K*_D_ values of this order are typical of specific protein-protein interactions. For example, *K*_D_ for mouse IgG anti-biotin antibody binding to biotin-BSA targets was found to be 2.3 nM [36], and binding of wheat germ agglutinin to epithelial cell glycoproteins has been reported as 0.32 μM [37]. By contrast, non-specific protein-protein interactions are considerably weaker, with *K*_D_ values in the millimolar range [32–34]. Interactions between nanoparticle corona proteins and cell surface biomolecules in cell culture would likely fall predominantly into the latter non-specific category, and cell surface adsorption of particles would therefore not be expected to significantly alter transport. Moreover, the same proteins constituting the ENM protein corona and participating in interactions with cell surface biomolecules, would also be highly abundant in free form within the media. To the extent that significant affinity existed between these proteins and cell surface biomolecules, the relatively abundant free protein molecules would occupy most of the available binding sites. In addition, we previously showed that when epithelial cells were incubated with metal oxide and silica-coated metal oxide ENMs [38] less than 0.1 % of the suspended material was either associated with or had transmigrated through cells after 24 h [18], consistent with non-specific binding and negligible effects of adsorption on transport. These data and considerations taken together suggest that a reflective boundary condition is most likely for suspensions of most industry relevant metal and metal oxide ENMs suspended in culture media. Nevertheless, further work is needed to fully assess the role of adsorption and uptake under various conditions and with various materials and cells. We have previously shown, for example, that in the absence of a protein corona (i.e. in protein-free media), metal oxide ENM agglomerates adhere more tightly to cell surfaces than they do when protein is present [21]. Such interactions between naked metal or metal oxide particles and cell surface proteins could conceivably obtain *K*_D_ values sufficiently small as to affect transport. Likewise, conformational changes in serum proteins caused by ENM adsorption could potentially increase their affinity for cell surface proteins, leading to greater binding and uptake of ENMs [39]. Further investigation to assess *K*_D_ for various ENM/cell systems are underway in our lab.

The DG model provides accurate modeling of transport for polydisperse suspensions by treating particle size as a pair of arrays (diameter and corresponding fraction of total mass), calculating transport of each size species separately, and summing the resulting mass concentrations over all species. As might be expected, and shown in Fig. 6, results obtained using polydisperse size distribution were in most cases substantially different from those obtained using a volume-weighted mean size. These differences were considerable for all materials examined, in spite of the relatively small polydispersity indices (PdI ≤ 0.3) measured by DLS for three of the four ENMs (Table 1). Although the differences for some materials at certain time points were negligible (e.g. CeO_2_ at 24 h and TiO_2_ at 12–15 h), it is clear that taking polydispersity into consideration improves accuracy of predicted dose metrics.

We should note that the effective density obtained by VCM and used in these simulations is an average density across all agglomerates, and it is likely that a distribution of effective densities is in fact superimposed upon the agglomerate size distribution. In addition, we have assumed that the composition and effective density of agglomerates are constant over the course of the simulated incubation. Although the close match between simulation and validation measurements presented here suggest that these approximations are reasonable, further studies to provide more detailed effective density characterization as a function of time and size are warranted.

In a recently-proposed dosimetry model, dynamic agglomeration of silver nanoparticles was estimated using stochastic modeling, based upon theoretical collision rates and estimated attractive forces [40]. Though promising, the detailed physicochemical characterization required for these estimates are not readily obtainable for the vast numbers of ENMs that require testing. Furthermore, we have previously shown that when ENM suspensions are prepared by sonication in DI water above critical delivered sonication energy (*DSE*_cr_), followed by dilution into culture media, the size distributions of the resulting suspensions are stable over time [11]. However, in cases where substantial concentration- or time-dependent changes in agglomeration state occur, corresponding adjustments to the model could easily be made, incorporating empirical measurements of agglomeration state over time and/or concentration, rather than relying upon theoretical estimates. Because of the iterative and compartmental design of the DG model, it is well suited to such modifications. Indeed, the associating protein system-transport modeling for which its antecedent was designed includes re-calculation of molecular species concentrations (based on stoichiometry, association constants and the law of mass action) at the end of each iteration [24–30]. An analogous approach could be employed to accommodate concentration or time-dependent changes in ENM agglomeration state.

The majority of ENMs are insoluble in aqueous solution, but when dissolution does occur, as it does with ZnO, this must be accounted for in dosimetry and transport modeling. Although it may be possible to theoretically estimate rates of dissolution over time in some specific cases [40], empirical measurements of ENM dissolution by ICP-MS, such as those reported by Xia et al. for ZnO [35], can readily be used to obtain dynamic dissolution data that can then be incorporated in to the transport model. In addition to decreasing solid ENM concentrations, solubilization may affect the size or distribution of sizes of agglomerates. The DG model allows specification of initial dissolution as well as either a constant dissolution rate or dissolution over a specified time to maximum fraction (Fig. 7). Beyond initial dissolution, assumed to have occurred prior to characterization of the suspension, further dissolution was modeled as a decrease in size of agglomerates corresponding to the decrease in mass. Additional studies to characterize size distribution over time for soluble materials will be helpful in refining the model for transport of such materials.

## Conclusions

In summary, both the CFD and the newly-developed DG models provide versatile and robust tools for accurately determining *in vitro* ENM concentration and deposition metrics over time. The DG model builds upon important earlier contributions in this area, allowing nanotoxicologists to account for polydispersity and solubilization of ENMs in suspension, as well as the effect of particle-cell binding at bottom of the well. Such advanced fate and transport models will enable nanotoxicologists to develop integrated and standardized dosimetric approaches, and will help to improve the accuracy and reliability of *in vitro* toxicological assays for engineered nanomaterials.

## Methods

### Nanomaterials and characterization.

ENMs investigated are listed in Table 1. SiO_2_, Fe_2_O_3_, and CeO_2_ ENM powders were generated in-house by flame spray pyrolysis using the Harvard Versatile Engineered Nanomaterial Generation System as previously described (VENGES) [41, 42]. TiO_2_ and ZnO ENM powders were obtained from EVONIK (Essen, Germany) and Alfa Aesar (Ward Hill, MA, USA) respectively.

Specific surface area, *SSA*, was determined by the nitrogen adsorption/Brunauer-Emmett-Teller (BET) method using a Micrometrics Tristar 3000 (Micrometrics, Inc., Norcross, GA, USA). Equivalent primary particle diameter, *d*_BET_, was calculated, assuming spherical particles, as

3 $$ {d}_{\mathrm{BET}}=\frac{6}{SSA\times {\rho}_{\mathrm{p}}} $$

where *ρ*_p_ is the particle density, which was obtained for each particle from the densities of component materials, at 20 °C, reported in the CRC handbook of Chemistry and Physics [43]. Particle crystal size and diameter was also determined by X-ray diffraction using a Scintag XDS2000 powder diffractometer (Scintag Inc., Cupertino, CA, USA), reported here as *d*_XRD_.

### ENM dispersal and characterization in suspension

Dispersions were prepared based on a protocol recently developed by the authors [11]. Briefly, sonication was performed in deionized water (DI H_2_O) using the critical dispersion sonication energy (*DSE*_cr_), which was determined as previously described for each ENM [11]. ENMs were dispersed at 5 mg cm^−3^ in 3 ml of solute in 15 ml conical polyethylene tubes, by sonication with a cup horn Branson Sonifier S450-A (Branson Ultrasonics Corporation, Danbury, CT) (maximum power output 400 W at 60 Hz, continuous mode, output level 3, power delivered to sample: 1.25 W). Stock DI H_2_O suspensions were then diluted to final concentrations in either RPMI + 10 % FBS or PBS + 0.5 % BSA and vortexed for 30 seconds. Dispersions were analyzed by DLS for determination of hydrodynamic diameters (*d*_H_) and polydispersity indices (PdI) using a Zetasizer Nano-ZS (Malvern Instruments, Worcestershire, UK).

### Effective density by volumetric centrifugation method (VCM)

Effective densities of ENMs were determined as previously described [12]. Briefly, 1.0 ml samples of 100 μg cm^−3^ suspensions of ENMs were dispensed into packed cell volume (PCV) tubes (Techno Plastic Products, Trasadingen, Switzerland) and centrifuged at 2,000 × *g* for one hour to pellet the suspended material in the bottom (capillary) of the PCV tube.

Agglomerate pellet volumes, *V*_pellet_, were measured using the *easy-measure* device from the PCV tube manufacturer. This device roughly resembles a thick steel ruler. The front face is etched along the top edge with graduations at 0.025 μl intervals from 0 to 5 μl. On the back face is a shelf that declines from near the top edge of the ruler at the left end, to near the bottom edge at the right end. The PCV tube rests with its bottom (capillary) end on the ramp, while being held in a sliding holder that wraps around the ruler. The front of the holder contains a lens that magnifies the view of the PCV tube capillary and the ruler graduation marks. The tube in its holder is slid along the ramp until the top edge of the pellet in the capillary is aligned with the top edge of the ruler, and the volume is read from the graduation mark coincident with that horizontal position.

Media densities were calculated from the mass of a 50 ml sample by subtracting the weight of a 50 ml volumetric flask from the weight of the same flask containing 50 ml of media. Effective agglomerate densities were calculated from *V*_pellet_ values of triplicate samples for each ENM, assuming a stacking factor, SF, of 0.634.

### Validation by frozen sections

Cylindrical receptacles for suspensions were created as follows. Polypropylene 15 ml conical tubes were cut down to approximately 5 cm in height and lined with polyethylene film. Approximately 3 ml of liquid paraffin wax was dispensed into each tube. A smooth 7 mm diameter hardwood dowel was inserted vertically into the paraffin in each tube, and maintained perpendicular to the bench surface until cooled, after which the dowel was removed, leaving a smooth cylindrical cavity of the same dimensions (7 mm in diameter, ~4 cm in height).

Nanomaterial suspensions were dispensed into the paraffin receptacles to create a column 10 mm in height (184 μl for the cylinder 7 mm in diameter), tubes were sealed with plastic wrap and incubated at 22 º C (the temperature employed for all simulations) for the designated times (24 h for TiO_2_, 48 h for CeO_2_ and 72 h for Fe_2_O_3_). Plastic-wrapped paraffin molds containing transported suspensions were carefully removed from tubes, flash frozen by immersing in liquid nitrogen for one minute, and moved to a pre-cooled (−30 °C) cryostat (CM-3000, Leica Biosystems, Buffalo Grove, IL, USA). Plastic wrap was removed and frozen paraffin broken away to extract the solid pellets of suspension, which were then mounted top side down in OCT fluid on specimen chucks. Pellets were sectioned at 25 μm and ten sections were combined per sample, representing a total thickness of 250 μm each. Samples were allowed to thaw and their ENM concentrations determined from optical absorbance and standard curves obtained with a NanoDrop 2000 UV–vis spectrophotometer (Thermo Scientific, Wilmington, DE, USA).

### Distorted Grid model

#### Input and Initialization

Input data required by the DG model include the initial mass concentration of the ENM, density of the bulk ENM material, effective density of agglomerates (e.g. from the VCM), height of the suspension column, the size or size distribution of the suspended particles (i.e. diameter and corresponding fraction of total solute for each particle species *j*), media density and viscosity, and temperature.

The vertical cylindrical column representing the cell culture well is divided by *n* + 1 horizontal boundaries into *n* compartments of equal height. The top and bottom of the well are the ‘impermeable’ boundaries 1 and *n* + 1, respectively; thus each compartment *i* is enclosed between boundaries *i* and *i* + 1. The initial distribution of each particle species *j* is described by assigning a value *C*_*i*,*j*_ to the mean concentration in each compartment. Since the initial suspension is homogeneous throughout the well, this is equal to the initial concentration *C*_0,*j*_ of species *j* in the well for all cells. Thus,

4 $$ {C}_{i,j}={C}_{0,j};\kern0.5em 1\le i\ \le\ n $$

#### Modeling diffusion

According to Fick’s first law a substance flows from a region of higher concentration to a region of lower concentration at a rate (per unit cross-sectional area) proportional to the magnitude of the concentration gradient at the boundary of the two regions:

5 $$ J=-D\frac{\partial C}{\partial z} $$

where *C* is the concentration (kg m^−3^), *z* is the position (m), and the proportionality constant *D* is the diffusion coefficient (m^2^ s^−1^), which is defined by the Stokes-Einstein equation (Eq. 2).

During a short time interval, Δ*t*, the mass of solute species j moving upward out of compartment *i*, and across boundary *i*, *M*_out_, can be represented as

6 $$ {M}_{\mathrm{out}}=A\Delta t{D}_{i,j}\frac{C_{i-1,j}-{C}_{i,j}}{\Delta z} $$

where *A* is the cross-sectional area of the column, *D*_*i*,*j*_ is the diffusion coefficient of particle species *j* at boundary *i*, *C*_*i* − 1,*j*_ and *C*_*i*,*j*_ are the concentrations of particle species *j* in the two compartments, and Δ*z* is the distance between the centers of the two compartments. Likewise, the mass of particle species *j* moving upward into compartment *i*, across boundary *i* + 1, *M*_in_, is given as

7 $$ {M}_{\mathrm{in}}=A\Delta t{D}_{i+1,j}\frac{C_{i+1,j}-{C}_{i,j}}{\Delta z} $$

These mass movements of material into and out of a compartment in single round of diffusion are depicted in Additional file 1: Figure S1 b. The net change in concentration of species *j* in compartment i, Δ*C*_*i*,*j*_ can be defined as

8 $$ \Delta {C}_{i,j}=\frac{M_{\mathrm{in}}+{M}_{\mathrm{out}}}{A\Delta z} $$

Substituting Eqs. 6 and 7 into Eq. 8, and simplifying gives

9 $$ \Delta {C}_{i,j}=\left(\frac{\Delta t}{\Delta {z}^2}\right)\left[{D}_{i,j}\left({C}_{i-1,j}-{C}_{i,j}\right)+{D}_{i+1,j}\left({C}_{i+1,j}-{C}_{i,j}\right)\right] $$

Note that the cross-sectional area *A* cancels and is thus not needed for this calculation. In each iteration of the model, Eq. 9 is applied to each compartment, and the new concentration of species *j* in compartment *i* after time Δ*t* is calculated as:

10 $$ {C}_{i,j}^{\hbox{'}}={C}_{i,j}+\Delta {C}_{i,j} $$

Although the diffusion coefficients for a given species *j* at each boundary *D*_*i*,*j*_ are considered identical for the systems modeled here, this may not always be the case. Here we have assumed that diffusion coefficients are independent of concentration and that possible cross-diffusion coefficients are negligible. However, the model has been implemented with an array of diffusion coefficients (one for each boundary) for each agglomerate species *j*, allowing future accommodation of more complex systems. The implementation and also includes an option to add a non-linear concentration dependence factor for the diffusion coefficient, represented as

11 $$ {D}_{i,j}^{\hbox{'}}=\frac{D_{i,j}}{1+k{C}_{i,j}}, $$

where *k* is the concentration dependence factor. For all simulations reported here the value of *k* was set to its default value of 0. We also assume for polydisperse suspensions that the concentrations of different species do not affect diffusion coefficients of one another, and that such systems can therefore be modeled by simultaneously but independently simulating transport of a each species. We further assume that particles or agglomerates do not dissociate and re-associate during transport, and that surface charge effects (attraction and repulsion) are negligible. However, the segmented, iterative design of the model would allow such considerations to be accommodated relatively easily, by adjusting concentrations and diffusion coefficients at the end of each short round of simulated transport.

#### Modeling sedimentation

The sedimentation velocity, *v*_s_, of a particle under gravity is defined by Eq. 1. The sedimentation coefficient of a particle, *S*, is defined as the ratio of a particle’s sedimentation velocity to the acceleration applied to it. Thus, for a particle sedimenting under gravity,

12 $$ S={v}_{\mathrm{s}}/g $$

where *g* is the acceleration due to gravity. The downward vertical displacement of a particle with sedimentation coefficient *S* in Δ*t* seconds is given by

13 $$ \Delta z=Sg\Delta t $$

In the previously-described method for simulation of velocity sedimentation that is the basis for this model [26–31], sedimentation was modeled by displacing compartment boundaries in the direction of sedimentation, and subsequently re-calculating solute concentrations in the bounded compartments. Because of this boundary migration the model did not adequately handle the situation at the bottom of the cell (bottom of the well in our model), since over the course of the simulation a number of boundaries would accumulate at the bottom of the cell and collapse associated compartments. In our model, since the concentration in the compartments at the bottom of the cell are of the most interest, it was necessary to revise the sedimentation component of the model so that solute (particles) could be moved between compartments without permanently moving compartment boundaries. This is accomplished by calculating, in each round of simulated sedimentation, the distance by which, for each particle species *j*, each compartment *i* would be displaced during the simulation time interval Δ*t*:

14 $$ \Delta {z}_{i,j}={S}_{i,j}g\Delta t $$

where Δ*z*_*i*,*j*_ is the downward displacement of compartment *i*, and *S*_*i*,*j*_ is the sedimentation coefficient of particle species *j* at the center (along the *z* axis) of compartment *i*. By the sedimentation of particle species *j* into compartment *i* from compartment *i* − 1 above, the concentration in compartment *i* is increased by the product of the concentration in compartment *i* – 1 and the fraction of the compartment height that is displaced into compartment *i*:

15 $$ \Delta {C}_{\mathrm{in}}={C}_{i-1,j}\left(\frac{\Delta {z}_{i-1,j}}{h}\right) $$

where *h* is the height of a compartment. Likewise, by sedimentation of particle species *j* out of compartment *i*, the concentration in compartment *i* is decreased by

16 $$ \Delta {C}_{\mathrm{out}}={C}_{i,j}\left(\frac{\Delta {z}_{i,j}}{h}\right) $$

The new concentration is thus calculated for compartment *i* by adding Δ*C*_in_ to, and subtracting Δ*C*_out_ from its previous concentration, which yields

17 $$ {C}_{i,j}^{\hbox{'}}={C}_{i-1,j}\left(\frac{\Delta {z}_{i-1,j}}{h}\right)+{C}_{i,j}\left(1-\frac{\varDelta {z}_{i,j}}{h}\right) $$

A single round of sedimentation for one compartment is depicted in Additional file 1: Figure S1b. In order to prevent compartment displacement in one iteration from completely overtaking the next compartment for the user-selected Δ*t*, the displacement that would be experienced by the particle species with the largest sedimentation coefficient in that time is calculated at the outset of the simulation. If that displacement is greater than *h*/2, then the largest time step allowable (producing displacement *h*/2) is calculated and used in place of the specified time step.

As with diffusion, although in the simulations described here it was assumed that the sedimentation coefficients at each boundary are identical, and that there is no dependence of sedimentation coefficients on concentration, the model is implemented with an array of sedimentation coefficients (one per boundary per species) and allows the introduction of concentration dependence. The sedimentation coefficient frequently varies in a non-linear way with concentration [44]. This relationship is often described by:

18 $$ {S}_{i,j}^{\hbox{'}}=\frac{S_{i,j}}{1+k{C}_{i,j}} $$

where *k* is a solute-dependent constant. For all simulations reported here the value of *k* was set to its default value of 0.

#### Iteration and output

The simulation proceeds by alternating rounds of diffusion (Eqs. 9 and 10) and sedimentation (Eq. 17) of duration Δ*t* until the selected cumulative time is obtained. At user-selected intervals during the simulation, as well as the end of simulation, the concentrations in each compartment, as well as derived mass, particle number and surface area dose metrics are exported to an excel file.

### Corrections to frictional coefficients

The DG model includes corrections to particle frictional coefficients in the form of ratios of corrected to ideal Stokes frictional coefficient, *f*/*f*^0^. Dividing the diffusion coefficients and sedimentation velocities by these ratios provides the associated correction for transport calculations.

As particle size approaches the mean free path of the media, *λ*, (≈ 2.5 × 10^−10^ m for water), slipping of solvent molecules at the particle surface (where ideally solvent has zero velocity) requires a correction referred to as the “slip”, or Cunningham correction factor, *C*_*c*_ [45, 46], such that:

19 $$ f/{f}^0=\frac{1}{C_c} $$

where

20 $$ {C}_c=1+\frac{\lambda }{d}\left(2.34+1.05{e}^{-\frac{0.39d}{2\lambda }}\right) $$

The magnitude of this correction for typical ENM particles and agglomerates is almost negligible (e.g. *C*_*c*_ ≈ 1.006 for *d* = 100 nm), and only becomes significant for particles with diameters <5 nm (*C*_*c*_ ≈ 1.12 for *d* = 5 nm, and *C*_*c*_ ≈ 1.30 for *d* = 2 nm). In the DG model this correction is applied by default unless otherwise specified by the user.

Additional corrections for non-spherical shape, solvation, and surface roughness can also be included. Whereas diameters obtained by DLS, being calculated from measured diffusion coefficients using the Stokes-Einstein equation (Eq. 2), are by definition hydrodynamic diameters (*d*_H_), which account for these effects, diameters measured by other methods (TEM, TRPS) may more accurately be considered diameters of idealized (unsolvated, smooth) spheres of equivalent volume (*d*_E_). A correction of the form *f*/*f*^0^ for non-spherical shape, commonly referred to as a dynamic shape factor, *χ*, can be specified in the model input either directly, or by indicating one of four specific shapes for which *χ* is known or can be calculated from known equations. These shapes include a cube, for which *χ* = 1.08 [45], and a prolate ellipsoid, oblate ellipsoid and circular cylinder with a ratio of major to minor axis length *P* = *a*/*b*, for which *χ* can be calculated as follows [44]:

21 $$ \mathrm{Prolate}\ \mathrm{ellipsoid}:\upchi = \frac{P^{-1/3}{\left({P}^2-1\right)}^{1/2}}{ \ln \left[P+{\left({P}^2-1\right)}^{1/2}\right]} $$

22 $$ \mathrm{Oblate}\ \mathrm{ellipsoid}:\chi =\frac{{\left({P}^2-1\right)}^{1/2}}{P^{2/3}\mathrm{t}\mathrm{a}{\mathrm{n}}^{-1}\left[{\left({P}^2-1\right)}^{1/2}\right]} $$

23 $$ \mathrm{Circular}\ \mathrm{cylinder}:\chi =\frac{{\left(2/3\right)}^{1/3}{P}^{2/3}}{ \ln (2P)-0.3} $$

In most cases these corrections are relatively small. For example, for a prolate ellipsoid, oblate ellipsoid and cylinder with *P* = 3, *χ* = 1.11, 1.10 and 1.27, respectively.

For other shapes and solvation and roughness factors, estimation of *f*/*f*^0^ is a complex topic beyond the scope of this paper. The DG model nevertheless provides options for user-specified shape, solvation and roughness correction factors.

### Calculation of volume-weighted hydrodynamic diameter distribution and average

The volume-weighted fraction, *f*_V,*i*_, for each diameter, *d*_H,*i*_, corresponding to the number fraction, *f*_N,*i*_, obtained from DLS were calculated as

24 $$ {f}_{\mathrm{V},i}=\frac{f_{\mathrm{N},i}{d_{\mathrm{H},i}}^3}{{\displaystyle {\sum}_i}{f}_{\mathrm{N},i}{d_{\mathrm{H},i}}^3} $$

and the volume-weighted average hydrodynamic diameter, 〈*d*_H_〉_V_, as

25 $$ <{d}_{\mathrm{H}}{>}_{\mathrm{V}}=\sum_i{f}_{\mathrm{V},i}{d}_{\mathrm{H},i} $$

### Calculation of particle number and surface concentrations

The effective density of an ENM agglomerate, *ρ*_EV_, which we can measure by the VCM method, can be expressed as a weighted average of the media and raw nanomaterial densities:

26 $$ {\rho}_{EV}={f}_{\mathrm{V},NM}{\rho}_{NM} + \left(1-{f}_{\mathrm{V},NM}\right){\rho}_{\mathrm{m}} $$

where *f*_V,NM_ is the volume fraction of the agglomerate consisting of the nanomaterial, *ρ*_NM_ is the density of raw nanomaterial, and *ρ*_m_ is the density of the media. Solving for *f*_V,NM_ yields

27 $$ {f}_{\mathrm{V},NM}=\frac{\rho_{EV}-{\rho}_{\mathrm{m}}}{\rho_{NM}-{\rho}_{\mathrm{m}}} $$

The volume concentration of raw nanomaterial, *C*_V,NM_, can be expressed as the product of *f*_V,NM_ and the volume concentration of the agglomerate, *C*_V,agg_, which is also equivalent to the mass concentration of raw nanomaterial, *C*_NM_, divided by its density:

28 $$ {C}_{\mathrm{V},\mathrm{N}\mathrm{M}}={C}_{\mathrm{V},\mathrm{a}\mathrm{g}\mathrm{g}}{f}_{\mathrm{V},\mathrm{N}\mathrm{M}}=\frac{C_{\mathrm{NM}}}{\rho_{\mathrm{NM}}} $$

Dividing through by *f*_V,NM_ yields

29 $$ {C}_{\mathrm{V},agg}=\frac{C_{NM}}{\rho_{NM}{f}_{\mathrm{V},NM}} $$

Dividing by the volume of a single agglomerate, *πd*_H_^3^/6 (assuming spherical shape) gives the agglomerate particle number concentration as

30 $$ {C}_{\mathrm{N},agg}=\frac{6{C}_{NM}}{\pi {d}_{\mathrm{H}}^3{\rho}_{NM}{f}_{\mathrm{V},NM}} $$

where *d*_H_ is the hydrodynamic diameter of the agglomerate. Multiplying this by the surface area of a spherical agglomerate, *πd*_H_^2^, yields the agglomerate surface area concentration,

31 $$ {C}_{\mathrm{S},agg}=\frac{6{C}_{NM}}{d_{\mathrm{H}}{\rho}_{NM}{f}_{\mathrm{V},NM}} $$

Finally, assuming that agglomerates are homogeneous (i.e. that primary nanomaterial particles are evenly distributed throughout the agglomerate), the fraction of agglomerate surface made up by nanomaterial surface is identical to the volume fraction of nanomaterial in the agglomerate, namely *f*_V,NM_, and the nanomaterial surface concentration, *C*_S,NM_ is the product of *C*_S,agg_ and *f*_V,NM_. Thus,

32 $$ {C}_{\mathrm{S},\mathrm{N}\mathrm{M}}=\frac{6{C}_{\mathrm{NM}}}{d_{\mathrm{H}}{\rho}_{\mathrm{NM}}} $$

### Implementation of the Langmuir isotherm adsorption

Binding of agglomerates implemented as a Langmuir isotherm adsorption process, in which the equilibrium dissociation constant, *K*_D_, is defined as

33 $$ {K}_{\mathrm{D}}=\frac{k_{\mathrm{d}}}{k_{\mathrm{a}}}=\frac{\left(1-\theta \right)\left[P\right]}{\theta } $$

where *k*_d_ and *k*_a_ are the rate constants for desorption and adsorption, *θ* is the surface coverage, defined as the fraction of surface sites occupied by adsorbed particles, and [*P*] is the molar concentration of particles [31]. Rearranging Eq. 33, the fraction of surface sites occupied, *θ*, can be expressed as

34 $$ \theta =\frac{\left[P\right]}{K_D + \left[P\right]} $$

To implement Langmuir isotherm adsorption in the DG model, at the end of each round of simulated diffusion and sedimentation, the particle (agglomerate) molar concentration [*P*] (mol L^−1^) in the bottom compartment is calculated from the particle (agglomerate) mass concentration *C*_*P*_ as

35 $$ \left[P\right]=\frac{10^{-3}\times {C}_{\mathrm{p}}}{N_A{\rho}_{\mathrm{EV}}\left(\frac{4}{3}\pi {r}^3\right)} $$

where *N*_*A*_ is Avagadro’s number, *ρ*_EV_ is the effective density (agglomerate density) and *r* is the particle radius. The fraction of surface sites occupied is then calculated from Eq. 34. The factor of 10^−3^ in the numerator provides conversion of units from moles m^−3^ to moles L^−3^. Because the nature and distribution of the surface adsorption sites are hypothetical in our model, we assume that the fraction of sites occupied is equivalent to the fraction of surface area occupied. Particles available for adsorption are limited to those present in the bottom simulation compartment with a height of Δ*z*. The fraction of bottom surface that can be covered by the particles within this compartment can thus be estimated as

36 $$ {\theta}_{\mathrm{avail}}=\Delta z{C}_{\mathrm{p}}{A}_{\mathrm{m}} $$

where *A*_m_ is the cross-sectional area per mass of particle:

37 $$ {A}_{\mathrm{m}}=\frac{3}{4r{\rho}_{\mathrm{EV}}} $$

The fraction of particles in the bottom compartment that are bound is then calculated as

38 $$ {F}_{\mathrm{b}}=\frac{\theta }{\theta_{\mathrm{avail}}}\mathrm{f}\mathrm{o}\mathrm{r}\frac{\theta }{\theta_{\mathrm{avail}}}\le 1.0,\mathrm{o}\mathrm{r}\kern0.5em {F}_{\mathrm{b}}=1.0\kern0.75em \mathrm{f}\mathrm{o}\mathrm{r}\frac{\theta }{\theta_{\mathrm{avail}}}>1.0 $$

The concentration (mg cm^−3^) of particle bound within the bottom compartment is then calculated as

39 $$ {C}_{\mathrm{b}}={F}_{\mathrm{b}}{C}_{\mathrm{p}} $$

and the concentration of free particle is

40 $$ {C}_{\mathrm{free}}=\left(1-{F}_{\mathrm{b}}\right){C}_{\mathrm{p}} $$

Since bound particles do not contribute to the concentration gradient driving diffusion, the concentration of free particles is used for calculating diffusion in the bottom compartments.

### Computational Fluid Dynamics model

For CFD mass transport predictions, the Eulerian–Lagrangian solution of the Navier–Stokes equation was used to model the fluid phase as a continuum, while treating the dispersed particles as a discrete phase, and tracking each particle in the Lagrangian coordinate system within the calculated flow field. Particle-to-particle interactions were neglected and it was assumed that the dispersed phase occupied a much smaller volume fraction than the fluid phase. The Eulerian–Lagrangian approach calculates the particle trajectories by integrating the forces acting on each particle in a Lagrangian reference frame [47]. In the corresponding force balance equation, the particle inertia is equal to the sum of other forces acting on the particle:

41 $$ \frac{d{u}_{\mathrm{p}}}{dt}={F}_D\left(\overrightarrow{u}-{\overrightarrow{u}}_{\mathrm{p}}\right)+\frac{\overrightarrow{g}\left({\rho}_{\mathrm{p}}-\rho \right)}{\rho_{\mathrm{p}}}+\overrightarrow{F} $$

Here, $\overrightarrow{F}$ is an additional acceleration term, $\overrightarrow{g}$ is gravitational acceleration, *ρ* is the fluid density, *ρ*_p_ is the particle density, $\overrightarrow{u}$ is the fluid phase velocity, ${\overrightarrow{u}}_{\mathrm{p}}$ is the particle velocity, and ${F}_{\mathrm{D}}\left(\overrightarrow{u}-{\overrightarrow{u}}_{\mathrm{p}}\right)$ is the drag force per unit particle mass, the general expression for which is

42 $$ {F}_{\mathrm{D}}=\frac{18\mu }{\rho_{\mathrm{p}}{d}_{\mathrm{p}}^2}\frac{C_{\mathrm{D}}Re}{24} $$

Here, *μ* is the fluid viscosity, *d*_p_ is the particle diameter, *C*_*D*_ is a drag correction factor, and *Re* is the relative Reynolds number, which is defined as:

43 $$ Re=\frac{\rho {d}_{\mathrm{p}}\left|{\overrightarrow{u}}_{\mathrm{p}}-\overrightarrow{u}\right|}{\mu } $$

In the model design employed in this work, laminar diffusion, Brownian motion and gravitational force were considered, while other forces, such as thermophoretic Force and Saffman’s lift Force were neglected. Because *d*_p_ < 500 nm, Brownian motion was included in the additional force term ($\overrightarrow{F}$) [48, 49], and the Stokes-Cunningham Drag Law, which accounts for non-zero relative velocity of fluid at the particle surface (“slip”), was chosen [50]. In this specific drag law, *F*_D_ is defined as:

44 $$ {F}_{\mathrm{D}}=\frac{18\mu }{\rho_{\mathrm{p}}{d}_{\mathrm{p}}^2{C}_c} $$

where the factor *C*_*c*_ is the Cunningham correction factor, as defined above (Eq. 20). Note that *C*_*c*_ in the denominator here replaces the *C*_D_*Re*/24 term in the general drag force expression (Eq. 42).

In this study, the simulation domain encompassed as a cylinder space with constant dimensions of 1.5 mm diameter and 1.0 mm height. To reduce the computational resources required, transport was modeled within a *π*/4 radian sector of the circular cylinder representing the cell culture well. A structured grid with a total of 3,586,000 elements was employed to represent the computational spatial domain. Grid independency analysis was carried out by varying grid numbers in all three dimensions. Performance at different grid aspect ratios, ranging from 1.0 to 3.0 was analyzed to minimize computational resources. For the final simulations, grid sizes of 2.5 μm and 5.0 μm were selected for *z* direction and *x,y* directions of Cartesian coordinates, respectively, resulting in the uniform grid aspect ratio of 2.0.

In the sector-shaped simulation domain, a symmetry boundary condition was employed for the cutting planes (flat vertical sector surfaces). All other surfaces, including the top, bottom and outer curved cylinder surfaces, were modeled as no-slip wall boundaries with zero roughness and a constant temperature of 22 °C. A reflective boundary condition was imposed at all walls for the discrete phase mode [47, 50], with a default constant value of 1.0 for both normal and tangential coefficient of restitution [47].

A three-dimensional structured mesh with the finite volume method and double precision option were utilized to discretize the computational domain, and to describe the mass and momentum transport for each cell. The SIMPLE algorithm was used to solve the pressure and velocity components. Spatial discretizations for momentum and energy equations employed the second-order upwind scheme. Time-dependent terms were interpolated using the implicit second-order interpolation scheme. All particles were injected at *t* = 0 and assigned an initial velocity magnitude of 0.0 m s^−1^.

## Additional file

Additional file 1: Figure S1. Distorted Grid model. Figure S2 Cryosection validation method. Figure S3 Volume size distributions from DLS. Figure S4 DG vs VCM-ISDD. (DOCX 2479 kb)
