# Supplementary material for: Advanced computational modeling for in vitro nanomaterial dosimetry
Source: Part Fibre Toxicol. 2015 Oct 24;12:32. doi: 10.1186/s12989-015-0109-1 (PMC4619515; doi:10.1186/s12989-015-0109-1)
Supplement: Additional file 1: Figure S1. — Distorted Grid model. Figure S2 Cryosection validation method. Figure S3 Volume size distributions from DLS. Figure S4 DG vs VCM-ISDD. (DOCX 2479 kb) [file 12989_2015_109_MOESM1_ESM.docx]

**Supplementary Figures**


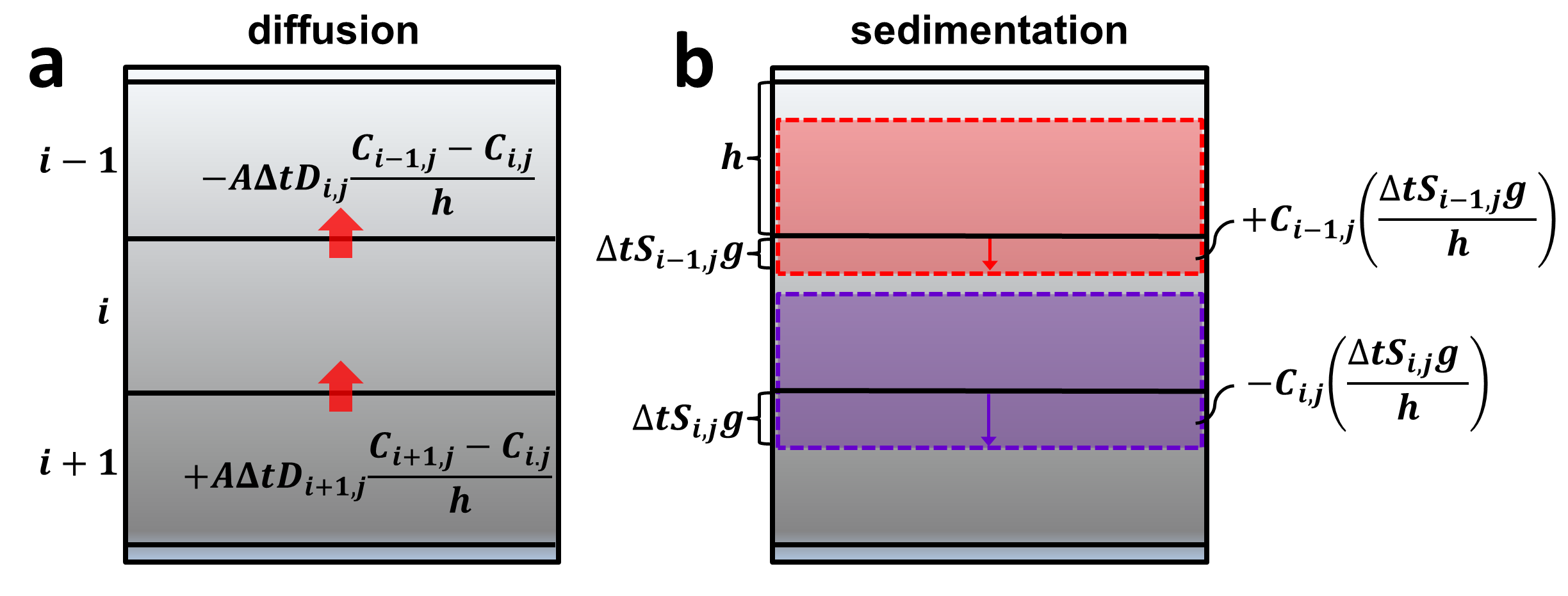


**Supplementary Figure 1 Distorted Grid model. a**, In a round of simulated diffusion, a new concentration for each particle species $j$ in each compartment $i$ is calculated from the mass concentrations of the particle in compartment $i$,($C_{i,j}$) the concentrations in the bounding compartments $i-1$ and $i+1$, ($C_{i-1,j}$ and $C_{i+1,j}$) the diffusion coefficient of the particle in compartments $i$ and $i +1$ ($D_{i,j}$ and $D_{i+1,j}$), the compartment height, $h$, simulated time step interval $\Delta t$, and cross-sectional area $A$ (Equations 6 and 7). **b**, In a round of simulated sedimentation, for each particle species, a displacement is calculated for each compartment determined by the particle sedimentation coefficient at boundaries $i$ and $i -1$ ($S_{i,j}$ and $S_{i-1,j}$), gravitational acceleration, $g$, and simulated time interval $\Delta t$ (Equation 14). A new particle concentration for each species in compartment $i$ is calculated by adding to the original concentration the product of the concentration in compartment $i-1$ and the fraction of compartment $i-1$ that is displaced into compartment $i$, and subtracting the product of the concentration in compartment $i$ and the fraction of compartment $i$ that moves into compartment $i+1$ (Equations 15 and 16).

**
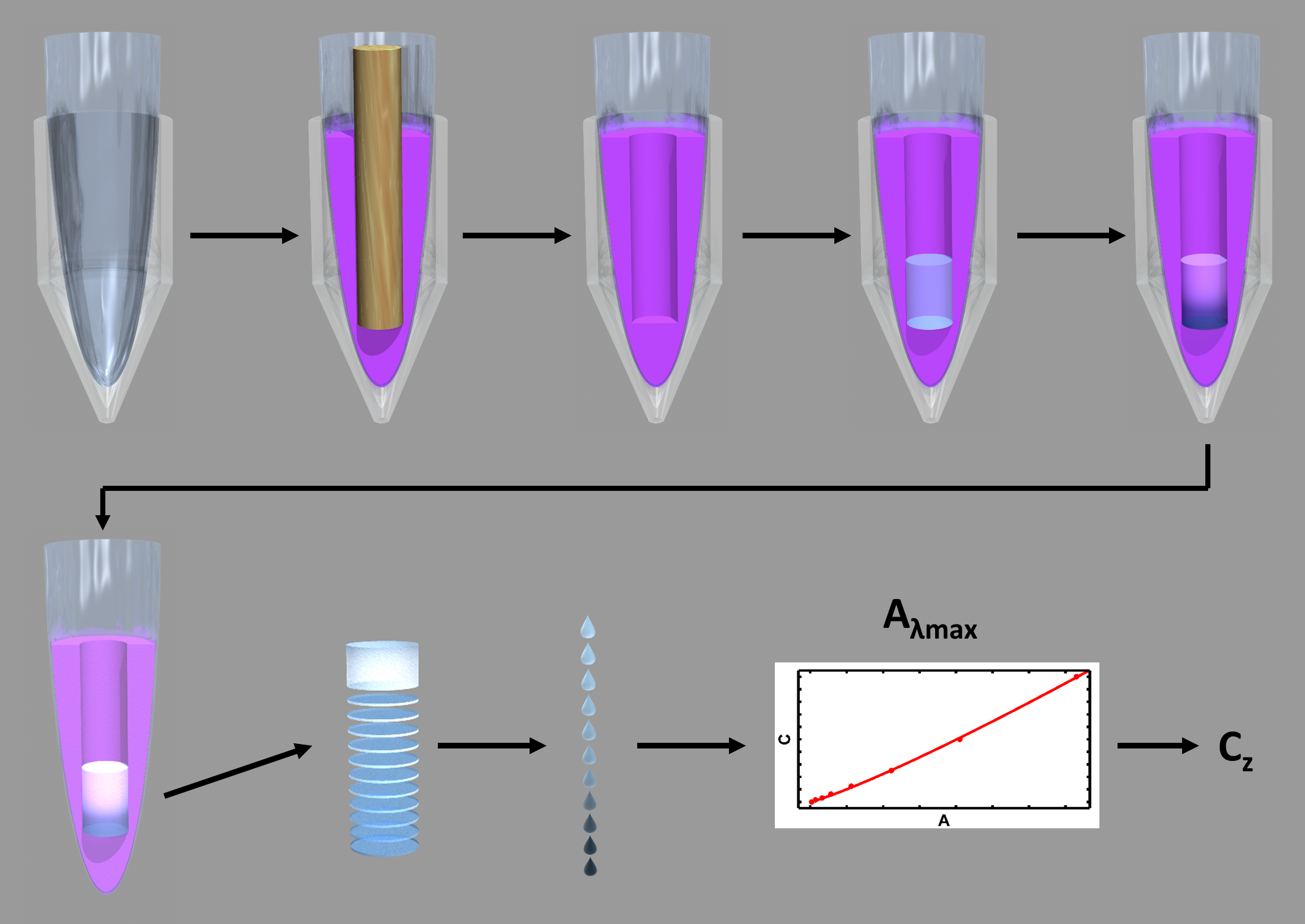
**

**Supplementary Figure 2 Cryosection validation method.** To experimentally validate the model a cylindrical paraffin cell was created as depicted here and described in methods. Particle suspension was added to the well. After allowing transport to proceed for selected lengths of time the well and suspension were flash frozen, and the frozen suspension pellet was extracted and cryosectioned. Thawed cryosections were analyzed by spectrophotometry and material concentrations in section samples were determined by spectrophotometry using a standard curve obtained at λ_max_ for the material/media combination.


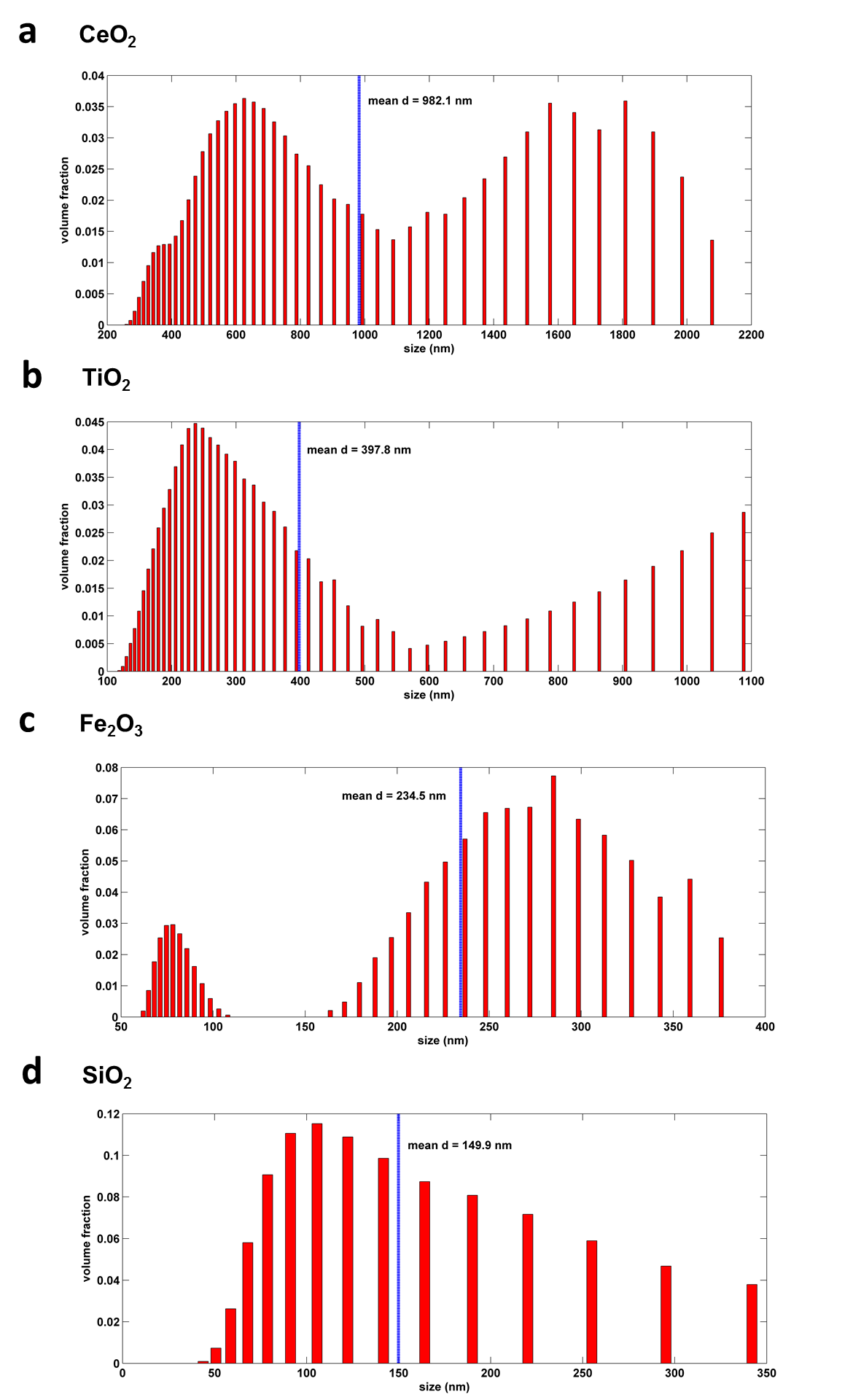


**Supplementary Figure 3 Volume size distributions from DLS.** Histograms distribution of agglomerate species size ($d_{\text{H}}$) distribution for volume size distribution from DLS.


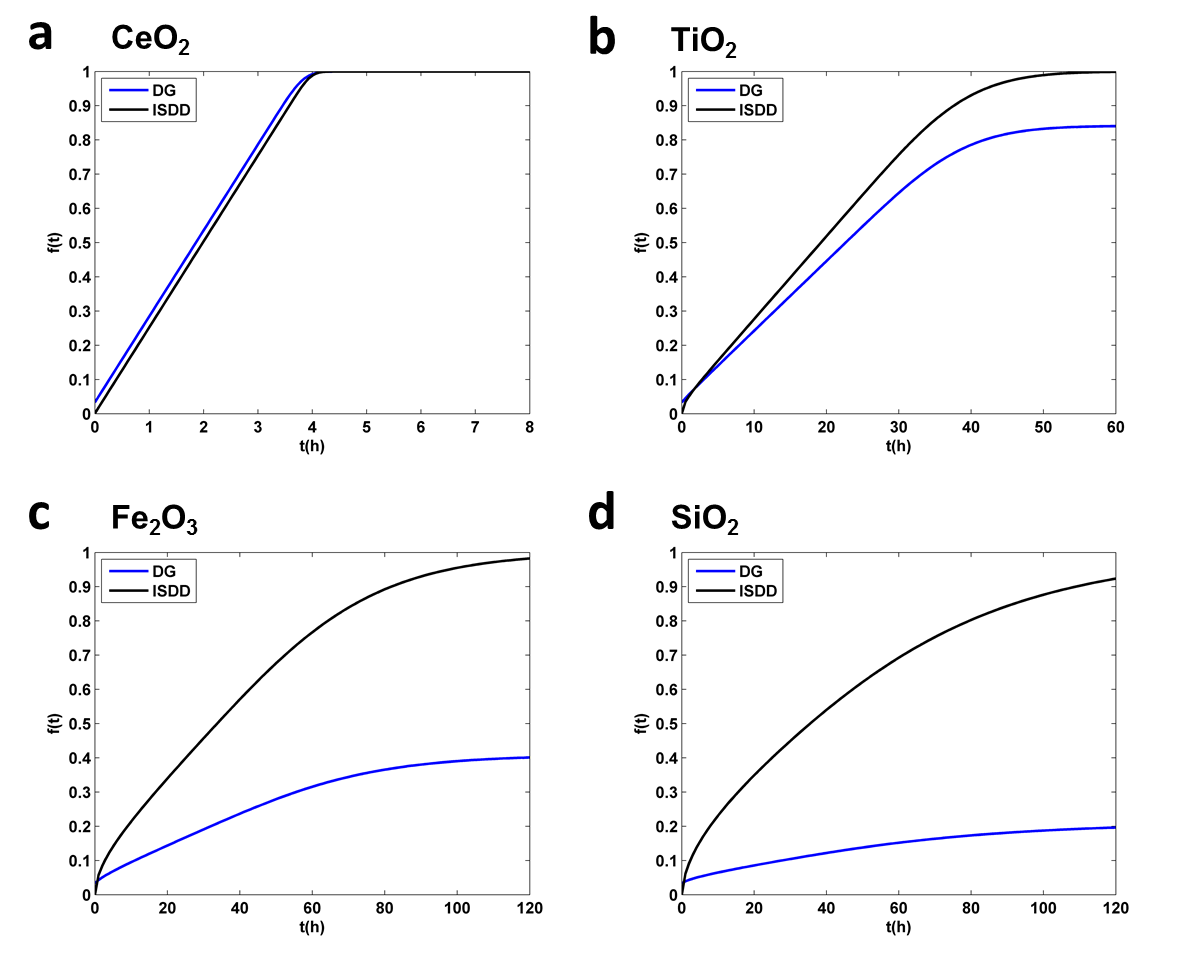


**Supplementary Figure 4 DG vs VCM-ISDD.** Comparison of deposited fraction predicted by DG and VCM-ISDD for SiO_2_, Fe_2_O_3_, TiO_2_ and CeO_2_ ENMs ($C_{0}$ = 0.1 mg ml^-1^, column height = 3 mm) with $d_{\text{H}}$= volume-averaged mean (Table 1). Distorted grid simulations performed assuming negligible particle-cell binding (reflective boundary condition), consistent with typical non-specific dissociation constants .
